# High diversity of genetic lineages and virulence genes in nasal *Staphylococcus aureus* isolates from donkeys destined to food consumption in Tunisia with predominance of the ruminant associated CC133 lineage

**DOI:** 10.1186/1746-6148-8-203

**Published:** 2012-10-29

**Authors:** Haythem Gharsa, Rym Ben Sallem, Karim Ben Slama, Elena Gómez-Sanz, Carmen Lozano, Ahlem Jouini, Naouel Klibi, Myriam Zarazaga, Abdellatif Boudabous, Carmen Torres

**Affiliations:** 1 Laboratoire des Microorganismes et Biomolécules Actives, Faculté des Sciences de Tunis, Université Tunis-El Manar, Tunis, 2092, Tunisia; 2 Departamento de Agricultura y Alimentación, Área de Bioquímica y Biología Molecular, Universidad de La Rioja, Madre de Dios 51, Logroño, 26006, Spain

**Keywords:** *Staphylococcus aureus*, Donkey, *spa* types, CC133, Virulence genes, Tunisia

## Abstract

**Background:**

The objective of this study was to determine the genetic lineages and the incidence of antibiotic resistance and virulence determinants of nasal *Staphylococcus aureus* isolates of healthy donkeys destined to food consumption in Tunisia.

**Results:**

Nasal swabs of 100 donkeys obtained in a large slaughterhouse in 2010 were inoculated in specific media for *S. aureus* and methicillin-resistant *S. aureus* (MRSA) recovery. *S. aureus* was obtained in 50% of the samples, being all of isolates methicillin-susceptible (MSSA). Genetic lineages, toxin gene profile, and antibiotic resistance mechanisms were determined in recovered isolates. Twenty-five different *spa*-types were detected among the 50 MSSA with 9 novel *spa*-types. *S. aureus* isolates were ascribed to *agr* type I (37 isolates), III (7), II (4), and IV (2). Sixteen different sequence-types (STs) were revealed by MLST, with seven new ones. STs belonging to clonal clomplex CC133 were majority. The gene *tst* was detected in 6 isolates and the gene *etb* in one isolate. Different combinations of enterotoxin, leukocidin and haemolysin genes were identified among *S. aureus* isolates. The *egc*-cluster-like and an incomplete *egc*-cluster-like were detected. Isolates resistant to penicillin, erythromycin, fusidic acid, streptomycin, ciprofloxacin, clindamycin, tetracycline, or chloramphenicol were found and the genes *bla*Z, *erm*(A), *erm*(C), *tet*(M), *fus*C were identified.

**Conclusions:**

The nares of donkeys frequently harbor MSSA. They could be reservoirs of the ruminant-associated CC133 lineage and of toxin genes encoding TSST-1 and other virulence traits with potential implications in public health. CC133 seems to have a broader host distribution than expected.

## Background

*Staphylococcus aureus* is a global problem for both humans and animals that affect hospitalized patients and also healthy individuals in the community. This microorganism is usually associated with skin and soft tissue infections. However, it is also able to cause serious diseases, such as pneumonia, meningitis, or septicaemia, among others. Additionally, the pathogenicity of *S. aureus* infections is facilitated by the expression of several virulence factors, which include cell wall-associated adhesins and several toxin groups [1,2]. The coordinated expression of these virulence factors is dependent on a global quorum-sensing regulator system, named *agr* (accessory gene regulator) [3]. Methicillin-resistant *S. aureus* (MRSA) is a well-recognized human pathogen that has also been identified as a veterinary and zoonotic pathogen. MRSA can cause animal infections and is also able to colonize the skin, nasal and oral mucosa of healthy animals. Several studies have reported the emergence of MRSA among farm animals [4-7]. Moreover, the emergence of MRSA in the equine population has been demonstrated [8-10] and, very recently, MRSA isolates have been detected in microbiota of healthy donkeys [11].

More than forty-four million donkeys do exist on our planet, of which 40 million (96%) inhabit in developing countries [12] where donkeys play an important role serving as draught animals (packing, carting, threshing, farm cultivation, riding) [12], as well as for feeding; this is the case of milk [13] or meat products [14]. In Tunisia, there are over a hundred twenty three thousand donkeys (http://www.onagri.tn), what represents over 65.5% of the total equine population and it is considered one of the countries with the largest number of donkeys [15,16]. Donkey food represents approximately 5% of total meat consumed in Tunisia and it is especially eaten in low income families (http://www.femmezoom.com/).

Few data do exist about the genetic lineages of *S. aureus* and the prevalence of MRSA that colonizes nares of healthy equids. So far, different clonal lineages such as CC8, CC22 or CC398 have been identified in horses [10,17]. Moreover, the isolates detected presented varied susceptibility to antimicrobials, different toxin profiles and diverse *agr* and SCC*mec* types depending on the clone found [10,17]. Remarkably, no *tst* and *lukF/lukS*-PV positive isolates have been detected in these animals; additionally, as far as we know, no previous studies of this type have been performed in Tunisia and there are very few reports in other African countries [12].

The objectives of this work were to analyse the prevalence of *S. aureus* in nasal samples of healthy donkeys destined for food consumption in Tunisia and to determine the genetic lineages and the presence of antimicrobial resistance and virulence genes in the recovered isolates.

## Methods

### Sampling and microbiological isolation

Nasal swabs from 100 healthy donkeys were obtained during March-May 2010 in a large slaughterhouse that receives animals destined for human consumption from farms of all Tunisia. The director of the abattoir gave his permission for taking the samples and it was supervised by the veterinarians of the abattoir. The ARRIVE (Animal Research: reporting of *in vivo* experiments) guidelines were followed in this study.

Nasal swabs were incubated in Tryptone Soy Broth (TSB) for 48h and then subcultured on Baird-Parker agar (BP) and ORSAB medium (Oxacillin Resistance Screening Agar Base, Oxoid) for 24–48 hours for *S. aureus* and MRSA recovery, respectively. Suspected *S. aureus* colonies were initially identified by conventional methods [Gram-staining, catalase test, oxidase test, DNase production, and ability to coagulate rabbit plasma (BioRad)]. *S. aureus* identification was confirmed by amplification of the species-specific *nuc* gene and, although all isolates were susceptible to methicillin, the presence of the *mecA* gene was also tested [5].

### Antimicrobial susceptibility testing

Susceptibility to 17 antimicrobial agents was performed using the disk-diffusion method [18]. Antimicrobial agents tested were (charge in μg/disk): penicillin 10 units, oxacillin (1), cefoxitin (30), kanamycin (30), gentamicin (10), tobramycin (10), tetracycline (30), chloramphenicol (30), trimethoprim-sulfamethoxazole (1.25/23.75), erythromycin (15), amikacin (30), ciprofloxacin (5), mupirocin (5), vancomycin (30), and teicoplanin (30). In addition, susceptibility to fusidic acid (10) and streptomycin (10 units) was carried out for which methods and breakpoints employed were those recommended by the Société Française de Microbiologie (http://www.sfm.asso.fr).

### Detection of antimicrobial resistance genes

The ribosomal methylases encoded by *erm*(A), *erm*(B) and *erm*(C) genes, which confer resistance to erythromycin and clindamycin, and the efflux pump encoded by *msr*(A) gene, conferring resistance to erythromycin, were studied by PCR in erythromycin-resistant isolates with primers and conditions as previously described [5]. In addition, *tet*(K), *tet*(M) and *tet*(L) genes, which confer resistance to tetracycline, *bla*Z gene to penicillin, *fus*B and *fus*C genes to fusidic acid, *ant*(6)-Ia, *str* and *ant*(3)(9)’ genes to streptomycin, and *cat*_*pC221*_, *cat*_*pC223*_, and *cat*_*pC194*_ genes to chloramphenicol were studied by PCR in antimicrobial-resistant *S. aureus* isolates [4]. Mutations in elongation factor G were studied by sequence analysis of *fus*A gene in all fusidic acid-resistant isolates [19].

### Molecular typing of *S. aureus* isolates

*Spa*-typing was performed in all *S. aureus* isolates as described elsewhere [20]. The polymorphic X region of *spa* gene was amplified by PCR, and sequences were analyzed using Ridom Staph-Type software version 1.5.21 (Ridom GmbH), which automatically detects *spa* repeats and assigns a *spa*-type according to http://spaserver.ridom.de/. Identification of *agr* allele group (I–IV) was determined by PCR as earlier described [21].

Multilocus-sequence-typing (MLST) was performed in selected *S. aureus* isolates (one isolate of each detected *spa*-type): the allelic profile of each isolate was obtained by sequencing internal fragments of 7 unlinked housekeeping genes (*arcC*, *aroE*, *glpF*, *gmk, pta, tpi,* and *yqiL*), allowing the determination of the sequence-type (ST), by the MLST database (http://saureus.mlst.net/). In these selected isolates, the clonal complexes (CC) were assigned according to the obtained ST; in the remaining isolates, the CC were assumed depending on the *spa*-types.

All isolates that presented *spa*-types associated with the clonal complex CC133 were tested for their capacity to coagulate bovine plasma (Sigma–Aldrich) following standard methodology [22].

### Detection of staphylococcal toxin genes

All isolates were tested by PCR for the presence of 18 genes coding for staphylococcal enterotoxins (*sea*, *seb*, *sec*, *sed*, *see*, *seg*, *seh*, *sei*, *sej*, *sek*, *sel*, *sem*, *sen*, *seo*, *sep*, *seq*, *ser* and *seu*), *tst* gene encoding the TSST-1 (Toxic Shock Syndrome Toxin) [23], *lukF/lukS*-PV genes encoding leukocidin PVL (Panton Valentine leukocidin), *lukED* genes encoding the bicomponent leukotoxin LukE-LukD, *lukM* gene coding for leukocidin M as well as *eta* and *etb* genes encoding exfoliative ETA and ETB toxins, respectively, and *hla*, *hlb*, *hld*, *hlg* and *hlg*_*v*_ genes encoding haemolysin toxins. The presence of all these genes were tested using primers and conditions as previously described [24].

## Results

### Field survey for *S. aureus* isolates from donkeys

No MRSA isolates were recovered in the 100 nasal samples of donkeys tested in this study when inoculated onto ORSAB plates. Nevertheless, *S. aureus* isolates were obtained from Baird-Parker agar plates in 50 of the 100 tested samples (50%), and one isolate per sample was further studied. The collection of 50 *S. aureus* isolates were cefoxitin and oxacillin susceptible, lacked the *mec*A gene, and then were confirmed methicillin-susceptible *S. aureus* (MSSA).

### Molecular typing of MSSA isolates detected in this study

The characteristics of the 50 MSSA isolates recovered in this study are shown in Table 1. Twenty-five different *spa*-types were detected, with nine of them being new and registered in the web site (http://spa.ridom.de/submission.shtml) as (number of isolates): t8449 (1), t8842 (1), t8840 (1), t8837 (1), t8836 (1), t7721 (1), t7720 (1), t7718 (2) and t7717 (2). The already described *spa*-types detected among our isolates were as follows (number of isolates when more than one): t1166 (14), t127 (4), t166 (4), t701 (4), t2484 (2), t091, t593, t1403, t1736, t1784, t2420, t3043, t3583, t3896, t4735, and t4781.

**Table 1:** **Phenotypic and genotypic characteristics of the 50 methicillin-susceptible *S. aureus *isolates recovered from healthy donkeys in Tunisia**

**Number of isolates**	***spa *type**^**a**^	**MLST**^**a b**^	**CC**^**b**^	***agr *type**	**enterotoxin genes detected**^**c d**^	**Other toxin genes detected**^**c**^	**Phenotype of antibiotic resistance**^**c,e**^	**Resistance genes detected**^**c**^
14	t1166	ST133	CC133	I	*see*^1^*, sei*^1^, *ser*^10^	*hla, hlb*^11^*, hld, lukED*	E^2^-FA^3^	*erm*(A)^2^
2	**t7718**	ST133	CC133	I	*see*^1^, *ser*	*hla, hld, lukED*		
1	t2420	ST133	CC133	I	*seq, ser*	*hla, hld, lukED*	E-FA	*erm*(A)
1	t1403	ST133	CC133	I	*ser*	*hla, hlb, hld, lukED*	P-E-S-FA	*bla*Z, *erm*(A)
1	**t8836**	ST133	CC133	I		*hla, hlb, hld, lukED*		
1	t3583	ST133	CC133	I	*seh*	*hla, hlb, hld, lukED*		
1	t4735	**ST2150**	CC133	I	*ser*	*hla, hlb, hld, lukED*		
1	**t8837**	**ST2111**	CC133	I	*sei, ser*	*hla, hlb, hld, lukED*		
4	t127	ST1738	CC1	III	*sea*^1^*, seb*^1^*, see*^1^, *sek*^1^*, seh, seq*^1^*, ser*^3^	*tst*^1^*, hla, hlb, hld, lukED,*	P^2^-FA^1^	*bla*Z^2^, *fus*C^1^
1	t1784	ST1	CC1	III	*seh, ser*	*hla,hlb,hld lukED*		
1	**t7720**	ST1	CC1	III	*seh, ser*	*hla, hld, lukED*	P-E-CP-FA	*bla*Z, *erm*(A)
1	t3896	ST1	CC1	III	*sea, see, seh, sek, seq*	*hla, hld, lukED*	P-FA	*bla*Z
4	t701	ST6	CC6	I	*sea*^3^*, see*^2^*, sed*^2^*, ser*^3^	*tst*^1^*, hla, hlb*^1^*, hld, lukED*	P-E^1^-S^1^-FA^1^	*bla*Z*, erm*(A)^1^, *erm*(C)^1^
1	**t8840**	ST6	CC6	I	*seh, seq, ser*	*hla, hlb, hld, lukED*	P	*bla*Z
4	t166	ST2057	CC522	I	*sec*^1^*, sej*^2^, *sel*^1^*, ser*^2^	*tst*^1^*, hla, hlb, hld, lukED, lukM*	E^3^-C^1^-FA^3^	*erm*(A)^3^, *erm*(C)^1^
2	**t7717**	ST72	CC72	I	[*seg, sei, sem, sem, seo, seu*]^1^*, see*^1^*, sel*	*hla, hlb, hld, lukED*		
1	**t7721**	ST72	CC72	I	[*seg, sei, sem, sem, seo, seu*]*, sec, sel*	*hla, hld, lukED*		
1	t091	**ST2110**	CC7	I	*ser*	*hla, hld, lukED*	P-TE-CP	*bla*Z, *tet*(M)
1	t593	ST15	CC15	II	*ser*	*hla, hld, lukED*		
1	t4781	**ST2181**	CC22	I	[*seg, sei, sem, sem, seo, seu*]*, ser*	*tst, hla, hld, hlg*	P	*bla*Z
2	t2484	**ST2151**	Singleton	II	[*sem, sen, sei, seu*]^1^*,sec, sel, sem*^1^	*tst*^1^*, hla, hlb, hld*		
1	t1736	**ST2109**	Singleton	IV	[*sem, sen, sei*]	*hla, hlb, hld, lukED*		
1	t3043	ST1660	Singleton	II	[*sem, sen, sei, seu*]*, sec, sel*, *ser*	*tst, hla, hlb, hld*	E	*erm*(A)
1	**t8449**	ST350	Singleton	I	[*sem, sen, sei, seu*]	*hla, hlb, hld*		
1	**t8842**	**ST2152**	Singleton	IV	*sej*	*etb, hla, hlb, hld, lukED*		

^a^ New *spa*-types or sequence types detected are shown in bold.

^b^ The MLST was performed in one isolate of each *spa*-type and the corresponding ST and CC were assumed for all isolates presenting the same *spa*-type.

^c^ In some cases, not all the isolates of the group presented the indicated characteristics (genes or phenotype). Whenever happened, the number of isolates with this characteristic is indicated in superscript.

^d^ Genes normally physically linked that compound the different *egc* clusters are included in brackets.

^e^ P: penicillin; E: erythromycin; TE: tetracycline; S: streptomycin; CP: ciprofloxacin; C: chloramphenicol; FA: fusidic acid.

Twenty-five MSSA isolates were typed by MLST (one isolate of each *spa*-type) and 16 different STs were identified, seven of them being new and registered as ST2109, ST2110, ST2111, ST2150, ST2151, ST2152 and ST2181 (Table 1). Two isolates showing new *spa*-types also presented new STs (ST2111-t8837 and ST2152-t8842). The STs or *spa*-types detected among our *S. aureus* isolates were found to be distributed within eight clonal complexes (% of the isolates): CC133 (44%), CC1 (14%), CC6 (10%), CC522 (8%), CC72 (6%), CC7 (2%), CC15 (2%), and CC22 (2%). The remaining detected STs (10% of the isolates) did not belong to any CC representing singletons. The new detected STs belonged to CC133 (2 isolates), CC7, and CC22, whereas three STs were singletons. All strains which presented *spa*-types associated with CC133 presented the ability to coagulate bovine plasma.

Amplification of the *agr* locus showed that *agr* group I was predominant (detected in 37 of 50 MSSA isolates, 74%), the remaining isolates being ascribed to *agr* group III (detected in 7 isolates, 14%), *agr* group II (4 isolates, 8%), and *agr* group IV (2 isolates, 4%).

### Characterization of antimicrobial resistance mechanisms and virulence genes

Sixty percent of *S. aureus* isolates showed susceptibility to all tested antimicrobial agents, while the remaining isolates revealed resistance to the following antimicrobials: penicillin (24% of isolates, carrying *bla*Z gene), erythromycin [16%, carrying *erm*(A) (8 isolates) and *erm*(A) + *erm*(C) (2 isolates)], tetracycline [2%, carrying *tet*(M) gene], and fusidic acid (24%, one strain carrying *fusC* gene). All 50 MSSA isolates showed susceptibility to amikacin, cefoxitine, ciprofloxacin, gentamicin, kanamycin, mupirocin, oxacillin, streptomycin, teicoplanin, tobramycin, trimethoprim-sulfamethoxazole, and vancomycin. No mutations in elongation factor G of *fus*A gene were detected in analyzed fusidic acid-resistant isolates.

Six MSSA isolates carried the gene *tst* encoding TSST-1 (12%). The gene *etb* encoding toxin ETB was detected in one isolate (2%). None of our isolates harbored the genes for PVL or ETA toxins. Other virulence genes carried by MSSA isolates were: *hla*, *hld* (100%), *lukED* (90%)*, hlb* (70%), *ser* (60%), *sei* (20%), *seh* (18%), *sen* (16%), *see*, *sem*, *seu* (14%), *sel* (12%), *sea*, *sec* (10%), *lukM*, *seq* (8%), *seg*, *sej*, *seo* (6%), *sed*, *sek* (4%), *seb, hlg, hlg*_*v*_ (2%) and the *egc* cluster-like [*sei*, *seg, sem, sen, seo, seu*](6%) (Table 1).

## Discussion

Very limited data on the nasal carriage of S. aureus in donkeys are available. In this sense, a study conducted in Ethiopia described the detection of *S. aureus* in the upper respiratory tract of 13% of tested donkeys [12]. Other study performed in Italy detected *S. aureus* in 6% of donkey milk samples [13]. The high recovery rate of *S. aureus* detected among nasal samples of healthy donkeys in the present report (50% of tested samples) is relevant, and represents the first study of this type in Tunisia. The high diversity of genetic lineages among the *S. aureus* recovered is noteworthy. However, it should be noted that all our strains were methicillin susceptible. Elevated clonal variety has been already detected in other studies among MSSA strains and it seems that MSSA of human show a higher genetic diversity than MRSA [25].

The 16 different STs identified by MLST among *S. aureus* isolates, with seven of them new, were distributed in eight clonal complexes (CC133, CC1, CC6, CC522, CC72, CC7, CC15 and CC22), and five singletons. Among these, CC133, CC1 and CC6 were predominant and grouped 68% of typed isolates.

Twenty two of our isolates (44%) corresponded to the major ruminant lineage CC133 [6,22], being the major detected lineage. Several previous studies have reported the existence of CC133 in clinical *S. aureus* isolates of cattle, goats and sheep [26-28]; nevertheless, this clone was not detected in an earlier study carried out in Tunisia on healthy sheep [29]. It is interesting to remark that the clonal complex CC133 was associated with different *spa*-types in donkey isolates in our study (t1166, t1403, t2420, t3583, t7718, t8836) or in sheep and goat isolates (t544, t2678, t3495, t4560, t5592, t7294, t7296, t7297, t7298, t7300) in previous studies [28]. It has been reported that *S. aureus* isolates of the clonal complex CC133 have been also responsible for most cases of mastitis in dairy farms [30], and it seems that this clone may have a broad geographic distribution. The *agr*-type I detected among the CC133 isolates in our study was coincident with former reports [31]. Some authors propose that strains of lineage CC133 could have evolved and adapted to small ruminants derived from humans due to an adaptive genome diversification resulted from allelic variation, gene loss, and horizontal acquisition of mobile genetic elements containing virulence genes with attenuated or enhanced activity in ruminants. The capacity to coagulate bovine plasma detected in our isolates belonging to this CC is a characteristic previously reported for isolates adapted to small ruminants and bovine [22]; and the acquisition of a novel staphylococcal pathogenicity island (SaPIov2) carrying a novel von Willebrand factor-binding protein (vWBP) with ruminant-specific coagulase activity has been described among isolates of this lineage [22]. On the other hand, a very recent study undergone in a Danish Zoo [32] has revealed that ST133 may have a broader host distribution since it was detected in *S. aureus* of a wide variety of animal species. However, to the best of our knowledge, this is the first description of this genetic lineage in donkeys [6].

Seven MSSA isolates were ascribed to the clonal complex CC1 (those of ST1 and ST1738) which is a common lineage among human isolates [26]. This data is in accordance with earlier reports [33] where *S. aureus* isolated from equines were more likely to cluster into human associated lineages. In the referred study performed in United Kingdom, equine-associated *S. aureus* isolates were assigned to the major human lineage CC1. Although this CC seems to be related to human isolates, a possible animal origin has also been suggested in other studies, given that its presence in other animal species is not exceptional [34].

The lineage CC6, less frequently detected among our isolates (5 isolates), has been previously detected in both human and animal *S. aureus* isolates [35,36]. Our CC6 MSSA isolates were classified into the *agr* group I, which is in harmony with previous studies [37].

Three MSSA isolates were ascribed to the clonal complex CC72; this clone has been detected in humans in different countries [38,39]. One isolate belonged to the clonal complex CC15, which has been before detected in humans at both community and hospital settings [40,41]. Another study conducted in five African countries [42] showed that 12% of *S. aureus* obtained from humans in the community corresponded to the CC15 clone. In our study, the characteristics of our CC15 isolate were different from other previous reports in relation to the *spa*-types, *agr*-types and virulence determinants [40,42].

One MSSA isolate was ascribed to lineage CC22, associated to the United Kingdom EMRSA-15, which is commonly isolated from pet animals, especially dogs [35,43]. This result is consistent with that of [17] where the MRSA isolates of equines belonged to the same clone EMRSA-15 (CC22) and harbored the *egc* cluster-like comprising the enterotoxin genes: *seg*, *sei, sem*, *sen*, *seo*, and *seu*[37].

Several virulence genes were detected in our MSSA isolates. The detection of the *tst* gene in 12% of isolates is interesting, although this percentage was lower than the one detected among *S. aureus* isolates from healthy sheep (78%) or healthy humans (20%) in Tunisia [29,44]. Nevertheless, the *tst* gene was not detected among *S. aureus* isolates of donkey milk in a previous report [13]. Most of our MSSA isolates harboured haemolysin genes, where the *hla* and *hld* genes were present in all our isolates. Similarly, a significant number of strains harboured the *hlb* gene, while the *hlg* and *hlg*_*v*_ genes were only present in one isolate. High occurrence of the *hld* gene in nasal *S. aureus* isolates of healthy sheep in Tunisia has also been reported [29]. Remarkably, most of our isolates (90%) carried the *lukED* genes, which are commonly present in cattle causing bovine mastitis infections [5]. In addition, a high number of enterotoxin genes, with 3 distinct *egc*-cluster-like variants, were observed. With this regard, some strains presented some but not all of the genes comprising the *egc*-cluster or *egc*-cluster-like. The absence of one or more genes in the *egc-*cluster has been previously reported [45,46]. Even though *S. aureus* associated food poisoning outbreaks are normally due to human isolates [47], the presence of a wide variety of enterotoxin genes in *S. aureus* from donkey reflects the adaptation of enterotoxigenic strains to different mammalian species. The presence of the exfoliatin *etb* gene in one isolate, which also revealed novel genetic characteristics (t8842-ST2152), is remarkable.

Most of our MSSA showed susceptibility to the antimicrobials tested (60%) with several exceptions. The low frequency of penicillin resistance detected among our isolates contrasts with the high frequency of this type of resistance reported for human isolates, even in commensal strains [44,45]; however, it is in agreement with isolates of sheep origin [29]. With regard to fusidic acid resistance, the rate found in different countries is very variable, with percentages ranging from <1% to >50% [48]. No mutations in elongation factor G have been detected in the *fus*A sequences of the analyzed fusidic acid resistant *S. aureus* isolates and the *fusC* gene was only detected in one strain. The resistance mechanism of the remaining fusidic acid resistant MSSA strains of this study remains unknown. In addition, 2 streptomycin- and one chloramphenicol-resistant isolates did not present any of the tested resistance genes.

## Conclusions

In conclusion, the nares of healthy donkeys could be a reservoir of *S. aureus* isolates of the small ruminant associated CC133 lineage and also of isolates carrying the toxic shock syndrome related gene *tst* and enterotoxin genes responsible for food poisoning outbreaks.

Remarkably, CC133 seems to have a broader host distribution having been detected in different animal species. More studies should be performed in the future to gain knowledge in the genetic lineages of S*. aureus* circulating among healthy animals, as well as in the capacity of these strains to produce virulence factors, due to the risk of animal-to-human bacterial transfer and the acquisition and dissemination of the *SCCmec* element, which is responsible for genetic background common to endemic methicillin resistant *S. aureus*.

## Abbreviations

MRSA: Methicillin-resistant *Staphylococcus aureus*; MSSA: Methicillin-susceptible *Staphylococcus aureus*; MLST: Multilocus-sequence-typing; ST: Sequence type; CC: Clonal complex; TSST–1: Toxic Shock Syndrome Toxin; PVL: Panton valentine leukocidin; ORSAB: Oxacillin resistance screening agar base.

Part of the results of this manuscript has been presented in the 7th European Congress on Tropical Medicine and International Health in Barcelona, Spain 3^rd^ -6^th^ October 2011.

## Competing interests

Non-financial competing interests do exist.

## Authors’ contributions

HG and RBS took the animal samples, isolated the *S. aureus* strains and identified them and carried out important part of the genetic analysis. They participated in the writing of the manuscript. KBS participated in the design of the study, writing of the paper and in the collection of samples and isolation of microorganisms. EGS and CL contributed to the genetic analysis of the virulence factors in the collection of isolates and in the revision of the paper. AJ and NK participated in the identification of *S. aureus* isolates and in the study of the resistance genes of the microorganisms. MZ contributed to the design of the study, the general discussion of the manuscript and the writing of the paper. CT and AB conceived the study, participated in its design and carried out the general coordination of it and helped to draft the manuscript. All authors read and approved the final manuscript.

## Authors’ information

EGS, CL, MZ and CT belong to the Research Group on -Antimicrobial resistance, virulence and molecular epidemiology in bacteria of animals, food, humans and the environment of the University of Rioja that is coordinated by CT (PhD in Pharmacy and professor of the University of Rioja) and with important contribution of MZ (PhD in Veterinary and Associate Prof. of the Univ. of Rioja). HG, RBS, KBS, AJ, NK and AB belong to the research group on Surveillance of antimicrobial resistance and virulence in animals, food and the environment and implication in human health of the Univ. of Tunis (in Tunisia), coordinated by AB (PhD in Biology and professor of that University).
